# Exploring the impact of the innovative compound 3-(3-(4-hydroxy-2-oxo-2*H*-chromen-3-yl)-5-(pyridin-3-yl)-1*H*-pyrazol-1-yl) indolin-2-one on accelerating wound recovery

**DOI:** 10.1038/s41598-026-37714-5

**Published:** 2026-02-21

**Authors:** Ahmed Sabt, Heba Abdelmegeed, Abdel-Razik H. Abdel-Razik, Mohamed G. Thabit, Marwa Balaha, Moataz A. Shaldam, Ahmed M. Reda, Ahmed A. F. Soliman, Mohamed Abdelraof, Nehad A. Abdel Latif, Mai M. Elghonemy, Eman Y. Ahmed, Mohamed A. Abdelrahman, Rasha Z. Batran

**Affiliations:** 1 Chemistry of Natural Compounds Department, Pharmaceutical and Drug Industries Research Institute, National Research Centre, Dokki, Cairo 12622 Egypt; 2 Department of Histology, Faculty of Veterinary Medicine, Beni-Suef University, Beni-Suef, 62511 Egypt; 3 Department of Pharmaceutical Chemistry, Pharos University in Alexandria, Canal El Mahmoudia street, Beside Green Plaza complex, 21648 Alexandria, Egypt; 4 Department of Pharmacy, “G. d’Annunzio” University of Chieti-Pescara, 66100 Chieti, Italy; 5 Department of Pharmaceutical Chemistry, Faculty of Pharmacy, Kafrelsheikh University, Kafrelsheikh, 33516 Egypt; 6 College of Pharmacy, University of Kut, Wasit, 52001 Iraq; 7 Department of Biochemistry, Faculty of Pharmacy, Egyptian Russian University, Badr City, Cairo 11829 Egypt; 8 Pharmacognosy Department, Pharmaceutical and Drug Industries Research Institute, National Research Center, Dokki, Egypt; 9 Microbial Chemistry Department, Biotechnology Research Institute, National Research Centre, 33 El Bohouth St, P.O.12622, Giza, Egypt; 10 Department of Pharmaceutical Chemistry, Faculty of Pharmacy, Egyptian Russian University, Badr City, Cairo 11829 Egypt

**Keywords:** Coumarin, Heterocyclic, Antibacterial, Wound healing, In vivo study, Molecular modeling, Biochemistry, Biotechnology, Chemistry, Drug discovery, Microbiology

## Abstract

Wound healing represents a significant challenge within the field of medical science. Contemporary clinical practices increasingly favor the utilization of herbal compounds to facilitate the repair process. Among these compounds, coumarin-a phytochemical noted for its antibacterial and wound-healing properties-has garnered considerable attention. This study investigates the potential advantages of incorporating coumarin into wound dressings within an experimental model. Our synthesized target compound 3-(3-(4-hydroxy-2-oxo-2*H*-chromen-3-yl)-5-(pyridin-3-yl)-1*H*-pyrazol-1-yl) indolin-2-one (**CPPI**) demonstrated notable antimicrobial activity against the pathogenic *Staphylococcus aureus* MRSA, *Bacillus cereus*, and *Pseudomonas aeruginosa*. Moreover, both in vitro and in vivo experiments showed that **CPPI** significantly enhanced the migration of skin fibroblast cells and promoted the wound healing process. Furthermore, it facilitated complete re-epithelialization of the wounds. Histological analysis revealed the formation of well-structured granulation tissue and a reduction in indicators of wound infection, evidenced by a minimal presence of inflammatory cells in comparison to untreated wounds. Additionally, in silico molecular docking studies of **CPPI** indicated significant binding affinity within COX-2 active site along with a stable complex during molecular dynamics simulations. Collectively, the findings of this study suggest that **CPPI** can provide a protective effect against infections in cutaneous wounds, attributable to its antimicrobial properties.

**Supplementary Information:**

The online version contains supplementary material available at 10.1038/s41598-026-37714-5.

## Introduction

The integumentary system plays a critical role in safeguarding the body against external threats. Any injury or damage sustained by living skin or tissue is categorized as a wound. Wounds can be classified into various types, including bruises, incisions, and cuts, based on the specific characteristics of the injury. A range of internal and external factors, such as disorders of local blood supply, thermal exposure, chemical agents, electrical currents, mechanical forces, and surgical interventions, can create adverse conditions that elicit diverse physiological responses within the organism^1^. Insufficient wound management may result in complications, including hemorrhage, infection, inflammation, scarring, and impaired angiogenesis besides tissue regeneration^2,3^. Consequently, it is imperative to apply wound dressings in a timely manner to protect the wound bed and facilitate the healing process by promoting effective regeneration without adverse consequences. Despite the development of numerous synthetic wound dressings that have been engineered using various polymers and bioactive ingredients, their physicochemical and biological properties remain constrained. This limitation presents a considerable challenge in enhancing their multifunctionality, particularly in terms of improving resistance to infection, neutralizing harmful free radicals, and ensuring optimal biocompatibility to promote rapid wound healing^4^.

Infections are recognized as a primary factor contributing to complications associated with wounds, particularly in environments conducive to the proliferation of microorganisms^5^. The emergence of multidrug-resistant infections further complicates the development of novel wound dressings that possess robust antimicrobial properties and facilitate effective healing^6^. Coumarin, a natural compound derived from tonka beans, licorice, strawberries, and apricots, displayed a broad spectrum of biological effects^7–13^, besides it has demonstrated significant potential in the context of wound healing^14^. Coumarin also exhibits antimicrobial properties, effectively inhibiting the growth of bacteria, viruses, and fungi^15–17^. Furthermore, coumarin functions as a potent antioxidant, safeguarding cells against oxidative damage and bolstering the body’s defense mechanisms. Consequently, these compounds promote the production of growth factors and stimulate angiogenesis, thereby enhancing blood supply to the wound and supporting improved tissue regeneration^18,19^.

**Fig. 1:**
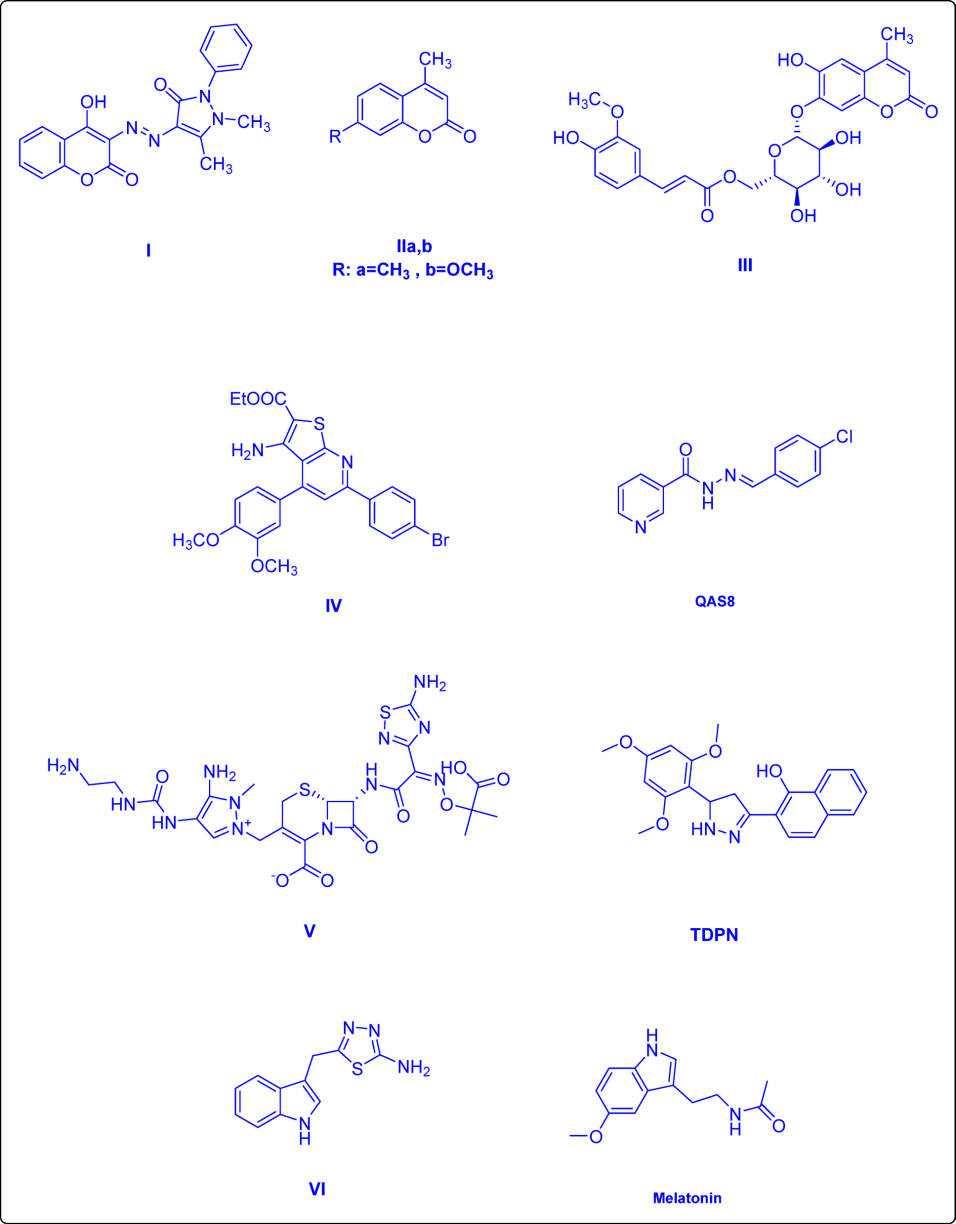
Compounds that possess antibacterial and wound healing activities.

The natural origin and low cytotoxicity of 4-hydroxycoumarin derivatives favor their potential in developing new antibacterial therapies due to their disruption of bacterial cell membranes and biofilm formation. For instance, researchers J. Sahoo and P. Sudhir Kumar synthesized a novel series of 4-hydroxycoumarin derivatives that are conjugated with arylazo groups. Their research revealed that most of these compounds exhibited significant antimicrobial and wound healing properties. Notably, compound **I** demonstrated considerable efficacy, with MIC of approximately 31.5 µg ml^−1^, and a higher level of tensile strength was observed in the experimental group compared to a control group^20^. Furthermore, FVA Dutra et al. reported the wound healing efficacy of two coumarin-derived compounds, specifically structures **IIa** and **IIb** which show considerable promise for enhancing wound repair^21^. Additionally, coumarin glycoside **III** isolated from the root of *Nymphoides peltata* exhibited high level of efficacy in wound dressing applications^22^. Equally, the importance of various heterocyclic compounds, such as pyridine^23,24^, pyrazole^25^, and indole moieties^26^, has been examined in relation to their efficacy as antibacterial agents and in wound healing. Pyridine-based compounds exhibited antibacterial activities, as exemplified by **IV**^27^, and demonstrated wound healing activities by influencing inflammatory responses and cellular processes necessary for tissue restoration. For example, N-alkylated pyridine-based organic salts, such as QAS8^24^, promoted wound closure for the in vivo models. As for the pyrazole-containing compounds, the fifth-generation cephalosporin ceftolozane **V** ^28^ showed more stability against β-lactamases due to the presence of the pyrazole ring, which contributed to the wound healing efficacy demonstrated by TDPN^29^, a compound that induced epithelial-mesenchymal transition in keratinocytes and enhanced wound closure through cell migration. Regarding the Indole-based compounds, methicillin-resistant *Staphylococcus aureus* has demonstrated a better response to the indole-thiadiazole **VI**^30^ than to ciprofloxacin, while melatonin treatment promoted wound healing and enhanced collagen synthesis, as indicated in Fig. 1^26,31,32^.

**Fig. 2:**
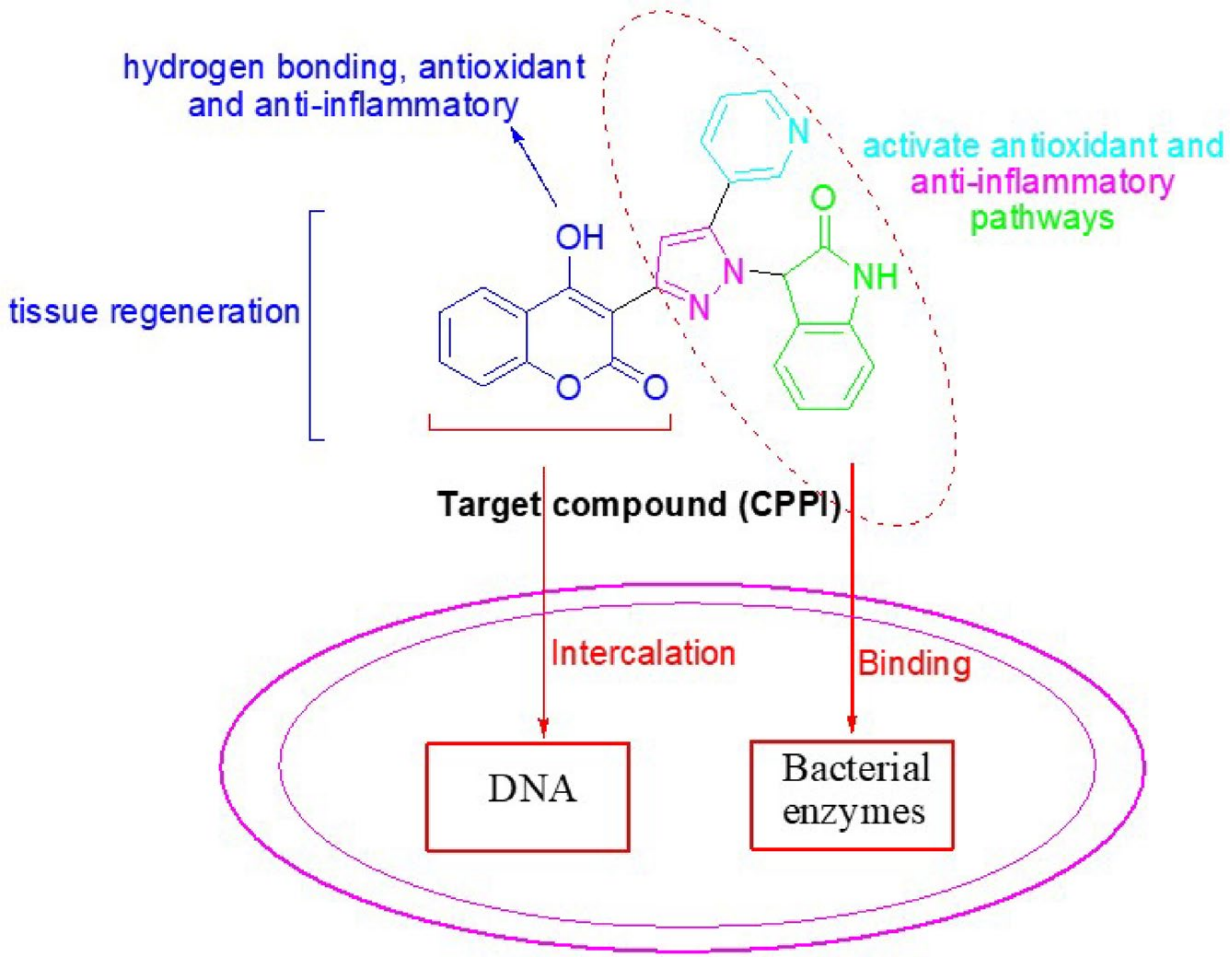
Rational design and common pharmacophoric features of antibacterial and wound healing agents.

In order to enhance the efficacy of wound dressings in the prevention of bacterial infections and to promote the wound healing process, a novel compound designated as **CPPI** has been synthesized. This compound features a coumarin core hybridized with heterocyclic scaffolds including pyridine, pyrazole, and indole moieties. As stated in literature^33–35^, the coumarin provides a rigid, planar structure crucial for binding to bacterial targets, which enables DNA intercalation and it contributes to tissue regeneration as well, while the C-4 hydroxyl group improves solubility, membrane penetration, hydrogen bonding and enhances antioxidant and anti-inflammatory activities, important in wound healing. Additionally, the nitrogen-containing heterocyclic moieties enhance binding to bacterial enzymes and activate antioxidant and anti-inflammatory pathways necessary in wound closure, Fig. 2. Molecular docking and dynamic simulation have yielded significant insights into the mode of binding, elucidating the interactions between **CPPI** and its target proteins. This interdisciplinary approach integrates theoretical chemistry with pharmacological evaluation, facilitating a comprehensive investigation of the properties and prospective applications of these compounds within the realm of drug discovery and development.

## Results and discussion

### Chemistry

**Scheme 1:**
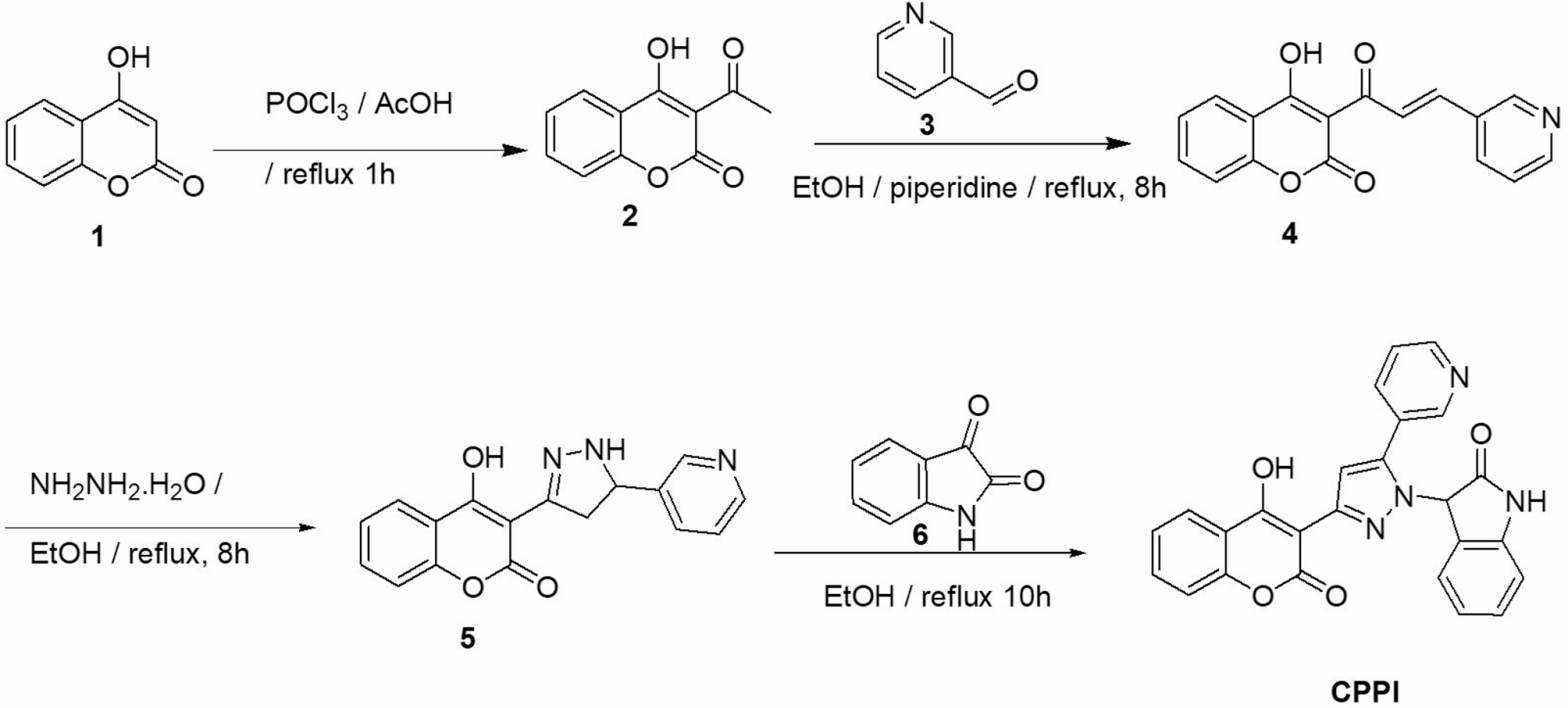
Synthesis of CPPI.

The synthesis of the target compound **CPPI** is outlined in Scheme 1. Initially, the acetylation of 4-hydroxycoumarin **(1)** with phosphorus oxychloride was carried out in glacial acetic acid, leading to the formation of 3-acetyl-4-hydroxycoumarin **(2)**. This intermediate subsequently underwent a Claisen–Schmidt condensation reaction with pyridine-3-carboxaldehyde **(3)** in ethyl alcohol and few drops of piperidine, resulting in the formation of the corresponding chalcone **(4)**. A cycloaddition reaction of the chalcone derivative **(4)** with hydrazine hydrate in ethyl alcohol yielded the respective pyrazoline (**5)**. The target compound, 3-(3-(4-hydroxy-2-oxo-2*H*-chromen-3-yl)-5-(pyridin-3-yl)-1*H*-pyrazol-1-yl)indolin-2-one **(CPPI)**, was synthesized through the condensation of the pyrazoline compound with isatin **(6)** in ethyl alcohol, which facilitated the elimination of a water molecule, as depicted in Scheme 1. The structural characterization of the target compound **CPPI** was achieved through elemental and spectral analyses, including, FTIR, ^1^H-NMR, ^13^C-NMR, and HR-TOF-ESI-MS. IR spectrum revealed stretching bands attributed to two carbonyl groups at 1722 and 1709 cm^−1^, in addition to OH absorption band at 3437 cm^−1^ and NH band at 3077 cm^−1^, respectively. ^1^HNMR spectrum displayed two singlet signals at 6.29 and 7.32 ppm assigned to the protons at position 3 of the indoline-2-one functionality and position-4 of the pyrazole moiety, respectively. Additionally, a singlet signal at 8.89 ppm corresponding to H-2 pyridine was clearly demonstrated. ^13^CNMR spectrum confirmed the structure of **CPPI** where the synthesized compound revealed the presence of signals of C-3 indoline at 61.12 ppm, C-3 coumarin at 95.38 ppm, C-4 pyrazole at 107.36 ppm and C-OH at 163.61 ppm, in addition to two signals corresponding to the carbonyl groups of indoline moiety and coumarin ring at 160.13 and 173.34 ppm, respectively. Furthermore, HR-TOF-ESI-MS spectrum showed a molecular ion peak at *m/z* 459.1069 [M + Na]^+^ that supported the structure of the synthesized compound.

#### Antimicrobial activity assessed through in vitro methods

Newly synthesized compounds which show significant potential as an antimicrobial agent can enhance wound healing as bacterial infections can impede this process^36,37^. To assess **CPPI** antimicrobial properties, we employed the agar cup plate diffusion method to examine Fig. S1. In this respect, we examined its effect against Methicillin-resistant *Staphylococcus aureus* (MRSA) (Gram-positive bacteria), *Bacillus cereus* (Gram-positive bacteria), and *Carbapenem-resistant Pseudomonas aeruginosa* (CRPA) (Gram-negative bacteria), as well as fungal pathogens such as *Candida albicans* and *Aspergillus niger*. As shown in Table 1, **CPPI** displayed significant antibacterial activity against both Gram-positive and Gram-negative bacteria, with the strongest activity observed against *Bacillus cereus*. However, **CPPI** showed no antifungal effect against *Candida albicans* and *Aspergillus niger*. As demonstrated in Table 2, The target compound exhibited an MIC of 30 µg/mL against *Staphylococcus aureus* (MRSA), which is lower than that of cephradine, polymyxin B, kanamycin, and ampicillin (MIC values: >100 µg/mL, 80 µg/mL, 40 µg/mL, > 100 µg/mL, respectively), indicating its superior potency. For *Bacillus cereus*, **CPPI** had an MIC of 20 µg/mL, which is more effective than cephradine, ampicillin, polymyxin B, and kanamycin (MIC values: 80 µg/mL, > 100 µg/mL, 30 µg/mL, 80 µg/mL, respectively). **CPPI** also showed an MIC of 30 µg/mL against *Pseudomonas aeruginosa* (CRPA), which is lower than the MIC values for cephradine, ampicillin, and kanamycin (> 100 µg/mL), suggesting **CPPI** is more effective than these antibiotics. In addition, the higher MIC value was also indicated for the targeted compound **CPPI** against fungal pathogens, particularly in case of *C. albicans*. Thus, the nature of this compound could be related to the bactericidal more than fungicidal activity. This finding was supported by the MBC and MFC investigation, Table 2, Which showed the significant activity towards bacterial pathogens (MBC less than 50 µg). On contrary, higher MFC (more than 200 µg) was clearly demonstrated, which reflected the lower antifungal activity of **CPPI** compound. Overall, these results indicate that **CPPI** exhibits superior antibacterial activity compared to cephradine, ampicillin, and kanamycin, but is less potent than ciprofloxacin, highlighting its promising potential as an antibacterial agent. Overall, the newly synthesized **CPPI** emerges as a promising antibacterial compound with potential applications in preventing wound infections, thereby enhancing wound healing. Our findings are consistent with previous studies that demonstrated the antibacterial activity of various coumarin derivatives^38–40^.

**Table 1:** Antibacterial activity of **CPPI** using agar cup plate diffusion method.

Compound	Inhibition zone (mm)				
	*Staphylococcus aureus* MRSA	*Bacillus cereus*	*Pseudomonas aeruginosa*	*Candida albicans*	*Aspergillus niger*
CPPI	4	5	4	ND	ND
Ciprofloxacin ^a^	5	6	4	–	–
Ampicillin ^a^	ND ^b^	3	ND ^b^	–	–
Polymyxin B ^a^	ND ^b^	2	3	–	–
Kanamycin ^a^	3	3	ND	–	–
Fluconazole	–	–	–	ND	3
Amphotericin B	–	–	–	4	5

^a^Ciprofloxacin, Ampicillin, Polymyxin B, and Kanamycin were utilized as the standard practice as antibacterial agents at 20 µg/mL. ^b^ND: not determined.

**Table 2:** Minimum inhibitory concentration (MIC) and minimum Bactericidal/Fungicidal concentration (MBC/MFC) of **CPPI**.

Compound	MIC (µg/mL)					MBC (µg/mL)				
	*Staphylococcus aureus* MRSA	*Bacillus cereus*	*Pseudomonas aeruginosa*	*Candida albicans*	*Aspergillus niger*	*Staphylococcus aureus* MRSA	*Bacillus cereus*	*Pseudomonas aeruginosa*	C*andida albicans*	A*spergillus niger*
CPPI	30	20	30	120	160	40	30	50	> 200	> 200
Ciprofloxacin	10	5	10	–	–	15	10	15	–	–
Cephradine	> 100	80	> 100	–	–	> 100	> 100	> 100	–	–
Polymyxin B	80	30	20	–	–	90	50	25	–	–
Kanamycin	40	80	> 100	–	–	60	90	> 100	–	–
Ampicillin	> 100	> 100	> 100	–	–	> 100	> 100	> 100	–	–
Fluconazole	–	–	–	80	20	–	–	–	100	30
Amphotericin B	–	–	–	10	5	–	–	–	15	10

#### In vitro evaluation of CPPI effect on cell migration

**Fig. 3:**
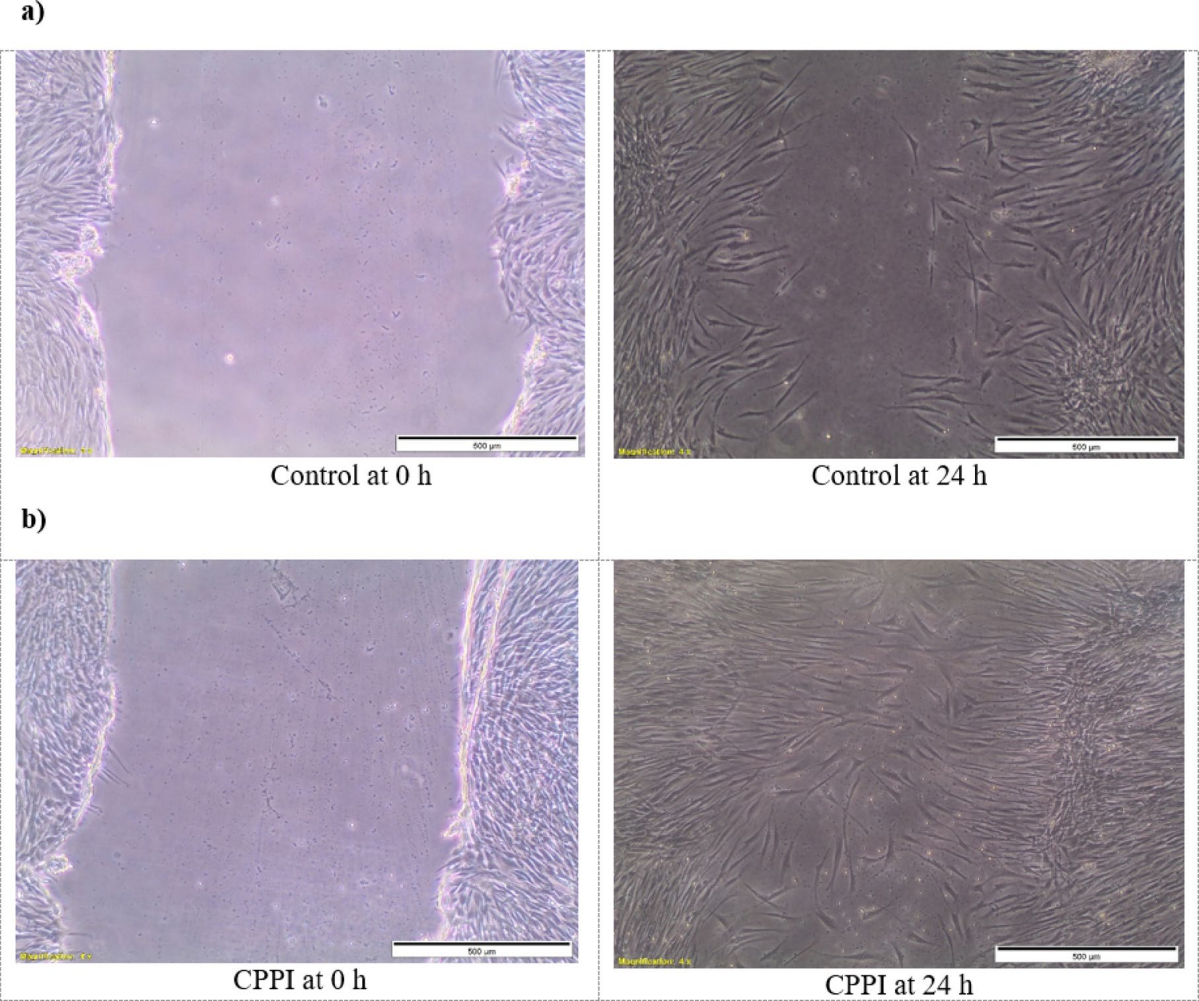
In vitro scratch wound assay for evaluation of cell migration for (**a**) untreated cells and (**b**) CPPI-treated cells at 0 and 24 h.

The impact of **CPPI** on human fibroblast cells was evaluated using a scratch healing assay in vitro. A monolayer of BJ cells was formed, a vertical scratch was made, and the cells were treated with **CPPI** for 24 h. As shown in Fig. 3, the CPPI-treated cells exhibited enhanced cell migration, resulting in a 91.1% closure of the scratch, compared to 71% closure in the untreated control cells. These findings suggest that **CPPI** promotes skin fibroblast cell migration and supports wound healing. Similar results for coumarin derivatives^22,41,42^ and pyridine nucleus^24,31,43 ^have been reported in several studies. These findings align with our results, highlighting the potential of coumarin and pyridine derivatives as effective wound healing agents.

#### In vivo assessment of CPPI effect on wound healing

**Fig. 4:**
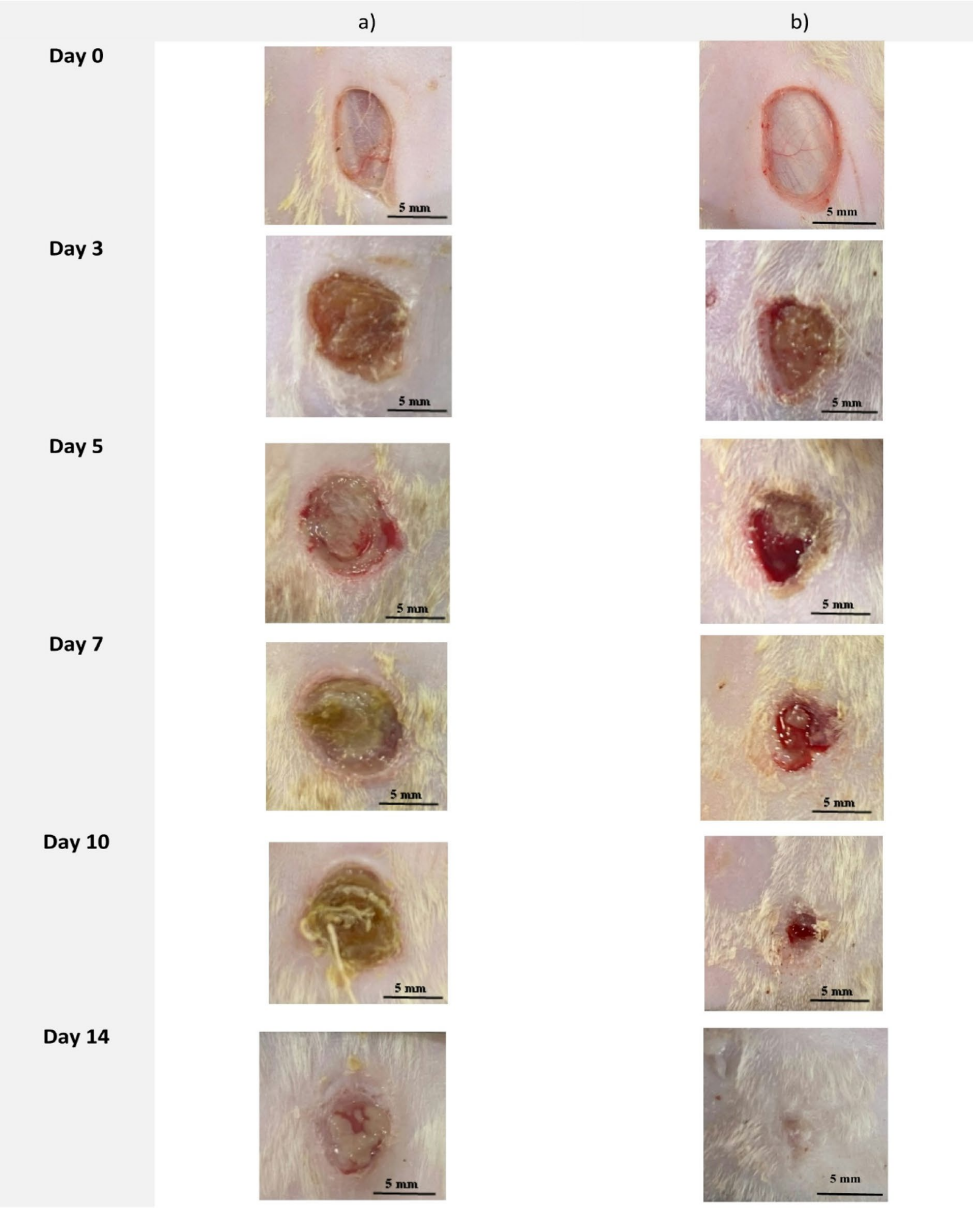
Representative images of the wound areas of the two groups (*n* = 5) showing the healing process: (**a**) No treatment, (**b**) **CPPI** as documented during a two-week timeframe.

**Fig. 5:**
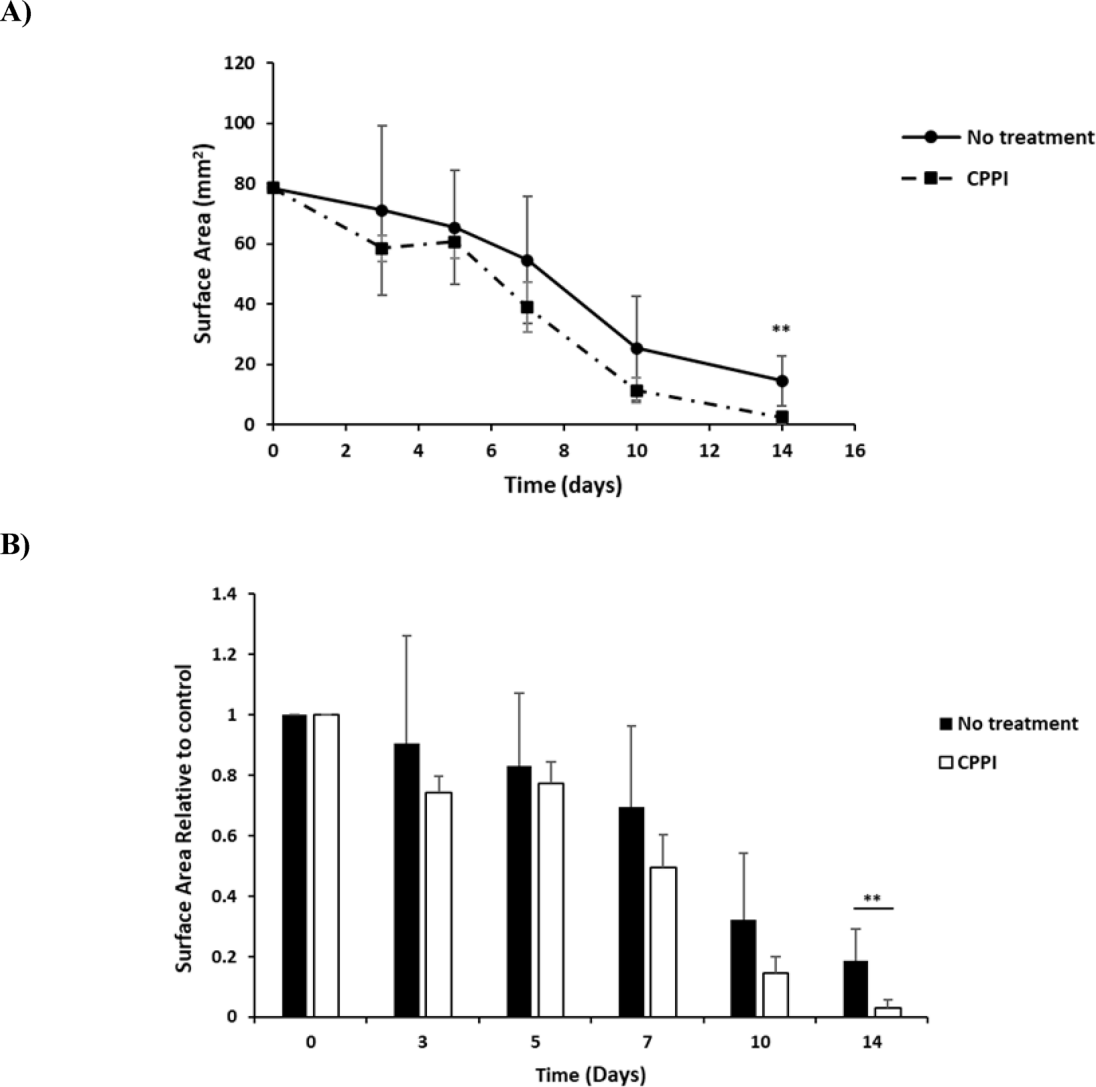
Alterations in wound size for both the untreated group and the group treated with **CPPI **were assessed over a span of two weeks. **CPPI** treatment significantly enhanced the wound healing process by the 14th day (*p*-value < 0.05). (**A**) The absolute wound surface area of both untreated and CPPI-treated groups which were monitored for two weeks. (**B**) The relative wound surface area of **CPPI** group compared to the untreated group which was monitored for two weeks.

Building on the previous results, we evaluated the potential of **CPPI** to enhance wound healing in vivo by topically applying the compound to skin wounds in rats and observing the effects on the wound healing process. Incisions were made on the dorsal region of two groups of rats, each consisting of five rats, with one group receiving no treatment and the other receiving **CPPI**. The wound-healing process was monitored over two weeks. Wound healing was assessed through systematic photography on the day of surgery (day 0) and on days 3, 5, 8, 10, and 14 post-operation to document the extent of wound closure. As shown in Fig. 4, by day 14, the untreated group showed limited epidermal growth toward the center of the wound, preventing full closure. In contrast, the CPPI-treated group exhibited nearly complete wound closure (96.8%) by day 14 (*p*-value < 0.05), with a significantly smaller wound area compared to the untreated group. This accelerated wound-healing process is further illustrated in Fig. 5A, B where Fig. 5A demonstrates the absolute wound surface area of both untreated and CPPI-treated groups while Fig. 5B demonstrates the relative wound surface area of **CPPI** group compared to the untreated group that was monitored over the period of two weeks. Together, these results highlight the significant effect of **CPPI** in promoting wound healing and in enhancing wound closure in vivo.

#### Histopathological assessment of wound healing

**Fig. 6:**
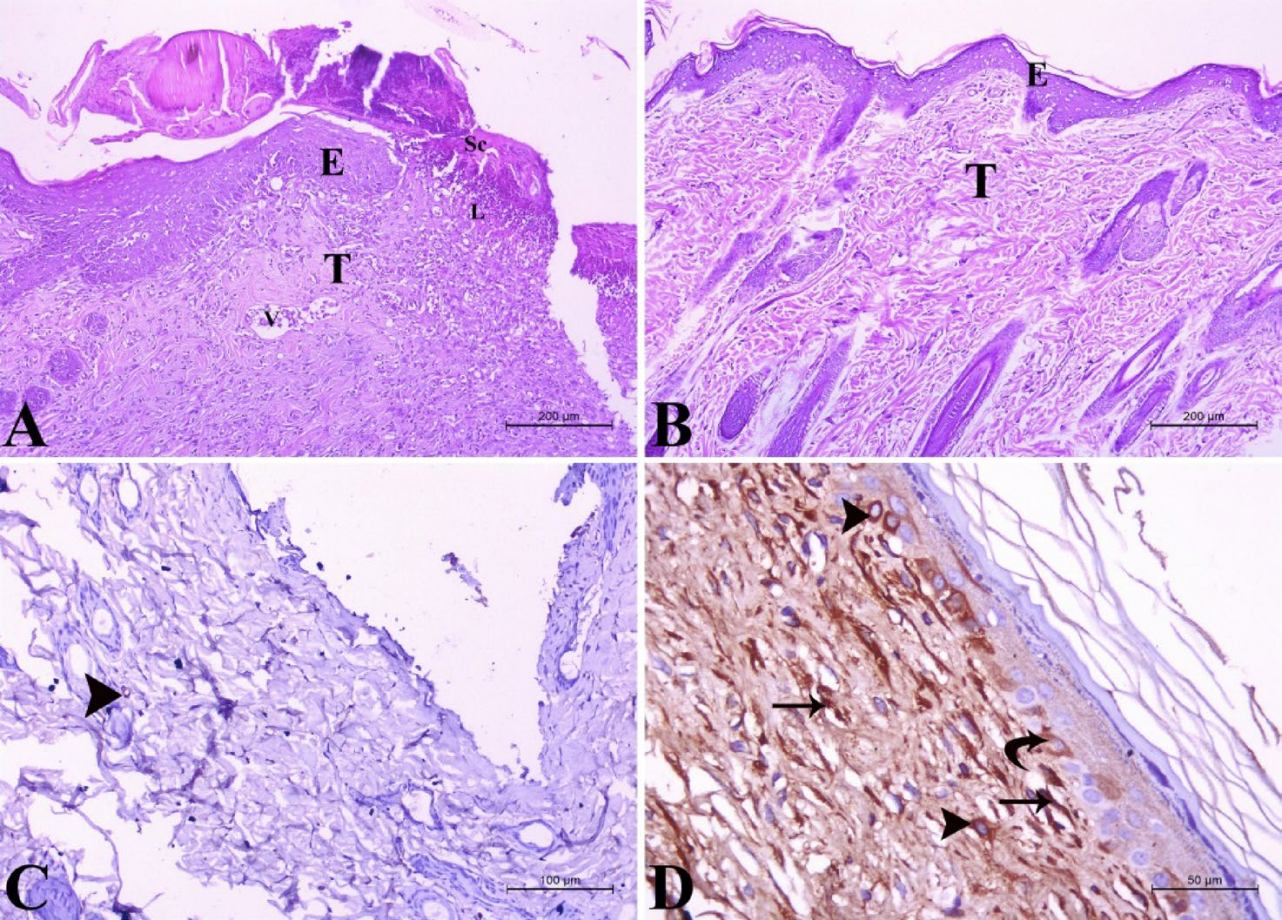
Histopathological examination of albino rat skin in the positive control and treated groups: (**A**) negative control group showing severe destruction of the epidermal (E) and dermal (T) layers, with total loss of the epithelial covering. Firmly attached scar tissue (Sc) is located above the destructed area. Severe inflammatory response in the affected area (L) is evident, along with the congestion of blood vessels (V). (H&E stain X100). (**B**) Treated group showing normal architecture of the dermal tissue (T) and epidermal tissue (E). (H&E stain X100). (**C**) Negative control group showing very faint expression of vascular endothelial growth factor in fibroblasts (arrowhead). (VEGF stain X200). (**D**) Treated group showing very strong vascular endothelial growth factor expression in the endothelial cells of blood capillaries (arrow), keratinocytes of the epidermis (curved arrow) and fibroblasts (arrowhead) (VEGF stain X400).

Histopathologically, the negative control group showed severe destruction and loss of the epidermal layers. The wound area was covered by firmly attached scar tissue with collagen fibers. The underlying dermal layers revealed massive fibrosis and severe inflammatory response in the wound area, with marked blood vessel congestion and extensive infiltration of neutrophils and lymphocytes. There was a very faint expression of vascular endothelial growth factor in fibroblasts Fig. 6A, C. On the contrary, treatment with the tested compound resulted in complete epithelization of the wound area, which was covered by a thick keratin layer. The underlying dermal tissue appeared normal, with newly formed blood vessels expressing vascular endothelial growth factor in the endothelial cells, as well as in the keratinocytes of the epidermis and fibroblasts Fig. 6B, D. The topical use of the tested compound on the wound accelerated the wound healing process showing epithelial layer thickening of 135.4 ± 1.35 μm and dermal layer thickening of 332.2 ± 2.67 μm when compared to the control group.

### Molecular modeling studies

#### Molecular docking

**Fig. 7:**
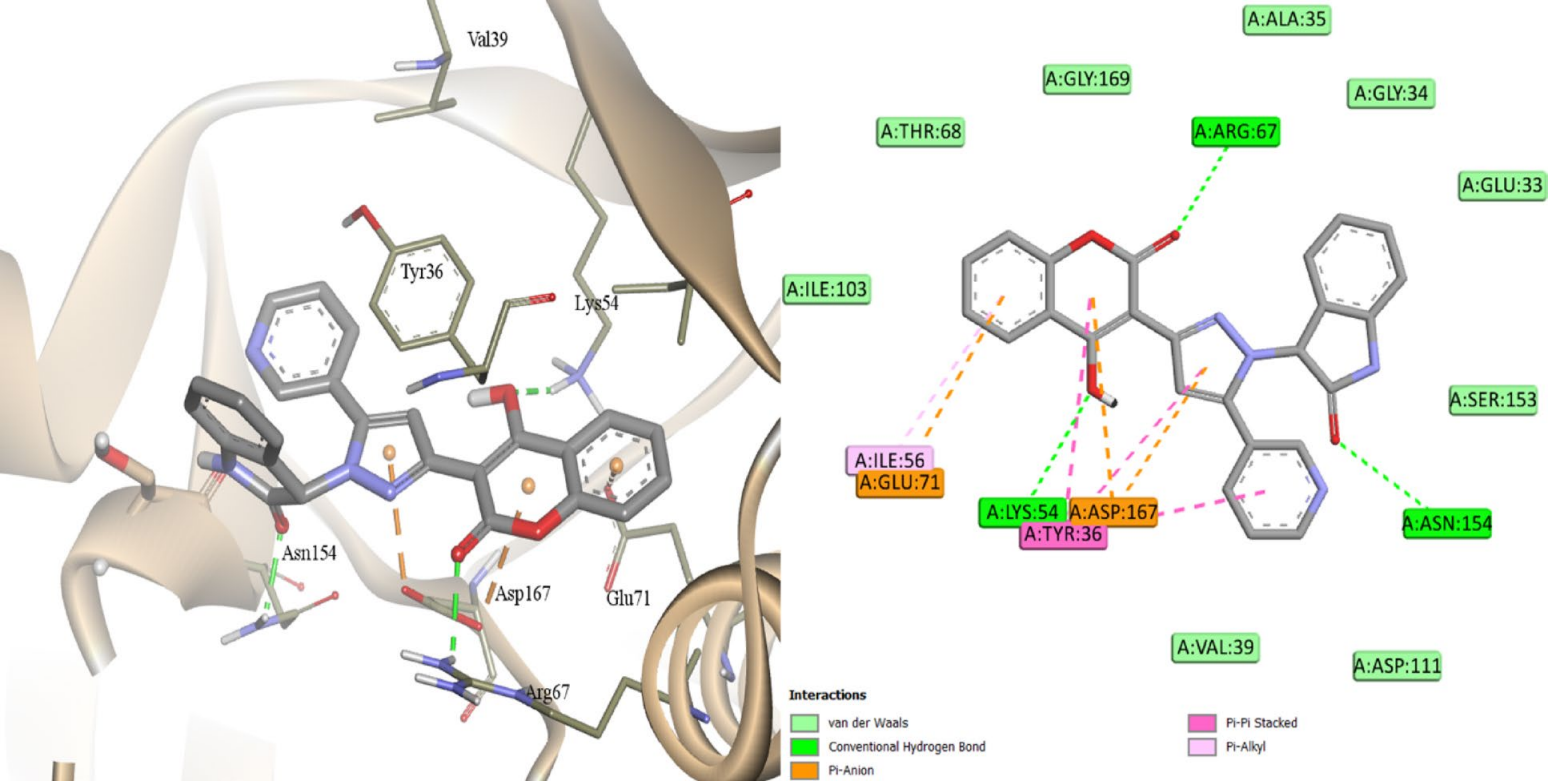
Docking of **CPPI** inside the active site of MAPK1 (PDB 4QTA^2^).

Molecular docking studies were performed to explain the therapeutic potential of **CPPI** through a screening approach against six target proteins associated with skin wounds (TNF-α, LOX, COX-1, COX-2, MAPK1 and MAPK3), and the binding patterns were analyzed based on the active site of the selected enzymes. Re-docking the co-crystallized ligand was the first step towards the docking process’s validation (Fig. S2). The best AutoDock score, and low bound energy were attributed to the best pose of the ligand-protein complex. The docking studies showed that MAPK1 followed by MAPK3 proteins have the highest affinity with **CPPI** ligand when compared with TNF-α, ALOX5, COX-1, and COX-2, Table 3, Figs. S3, S4. Our findings corroborate the action of coumarins as inhibitors of the mitogen-activated protein kinase (MEK), particularly MAPK1 (known as ERK2) and MAPK3 (known as ERK1)^44^ inhibitors and point out that these are promising molecular targets for tissue regeneration. In fact, **CPPI** formed a more stable complex with MAPK1 than MAPK3 (docking scores were − 11.4 kcal.mol^−1^, − 10.5 kcal.mol^−1^, respectively). **CPPI** forms three hydrogen bonds with the amino acid residue Arg67, Lys54 and ASN154 of MAPK1 through the carbonyl of coumarin, hydroxyl group and the carbonyl of indolone of the ligand, respectively, Fig. 7.

**Table 3:** Docking scores of **CPPI** and the cocrystal ligands with the different target proteins.

Target protein	Docking score	
	CPPI	Ref
MAPK1	− 11.4	− 14.4
MAPK3	− 10.5	− 14.5
TNFα	− 9.8	− 8.5
COX-1	− 9.2	− 10.6
LOX	− 9.1	− 7.1
COX-2	− 9.0	− 8.9

Furthermore, Tyr36 exhibited π-π stacking interaction with the coumarin and the pyrazole rings of **CPPI**, whereas Glu71 and the coumarin moiety’s phenyl ring exhibited π-anion interaction. The pyrazole ring’s aryl substituent interacted with Cys166 *via* π-sulfur. Regarding the other protein targets, **CPPI** established three hydrogen bonds with COX-1 (Tyr385, Ile523, and Ala527) and just one with TNF-α (Tyr151) and COX-2 (Arg120). **CPPI** did not create hydrogen bonds inside the MAPK3 and LOX active sites. Van der Waals, π–π stacking, π-sulfur, π-anion, π-cation, and π-sigma contacts were among the variable hydrophobic interactions that were noted. It was shown that **CPPI** showed almost equal affinity for LOX, TNF-α, and COX-1 and a significantly higher affinity for MAPK1 than MAPK3 as shown by the docking scores. Furthermore, **CPPI** engaged with the residues Tyr385, Ser530, Leu352, ALA527, Val523, Val349, and Tyr355 in a manner similar to that of the co-crystallized indomethacin derivative. Therefore, it was thought that **CPPI** would mainly operate through interaction with MAPK-1 rather than the other target proteins.

#### Molecular dynamic simulation

**Fig. 8:**
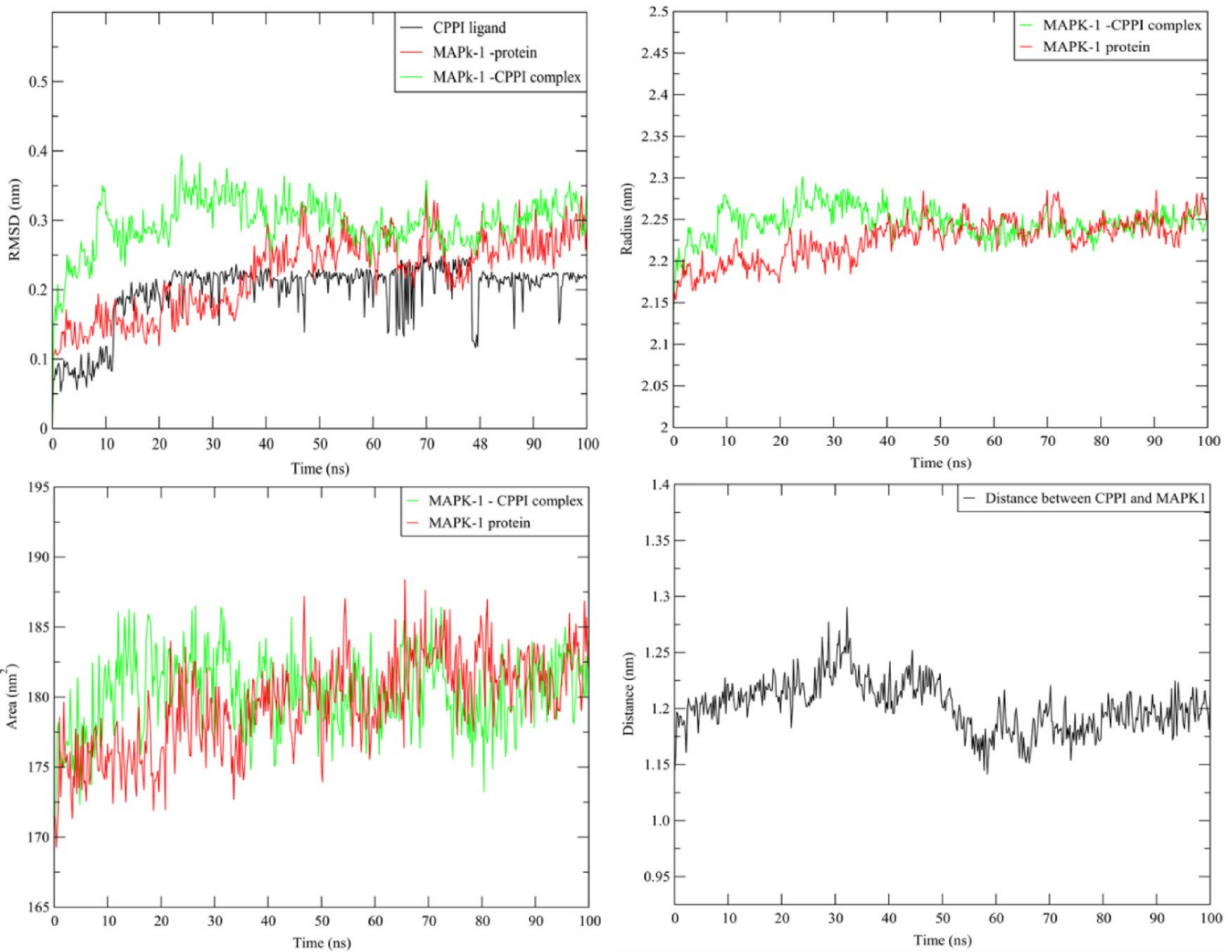
Plots of ligand-receptor distance, SASA, radius of gyration RMSD and RMSF for MAK1 protein with and without **CPPI**.

To investigate the stability of the **CPPI**-MAPK1complex, MD research was conducted on the optimal position of pose within the MAPK1 protein’s active area (binding cavity). Consequently, a 100 ns MD simulation carried out at room temperature was used to evaluate and compare the **CPPI** complex system with the apoprotein. First, the complex system’s convergence was confirmed by the simulation’s consistent temperatures, constant potential energy, and constant pressure Fig. S4. The difference between the MAPK1 protein in the presence and absence of compound **CPPI** was investigated by tracking the drift of RMSD along the trajectories. Figure 8 showed a similar pattern between the complex RMSD values and the apoprotein due to the stabilization of the MAPK1 protein. Furthermore, it was evident that the complex’s SASA and radius of gyration resembled those of the apoprotein, suggesting that no conformational changes were induced and that a stable complex with **CPPI** was formed. Additionally, the average distance of the CPPI/MAPK1 protein remained constant during the MD. Furthermore, the stability of the CPPI-MAPK1 protein complex was further supported by the lower draft in the complex’s RMSF compared to the free MAPK1 protein Fig. 8.

**Fig. 9:**
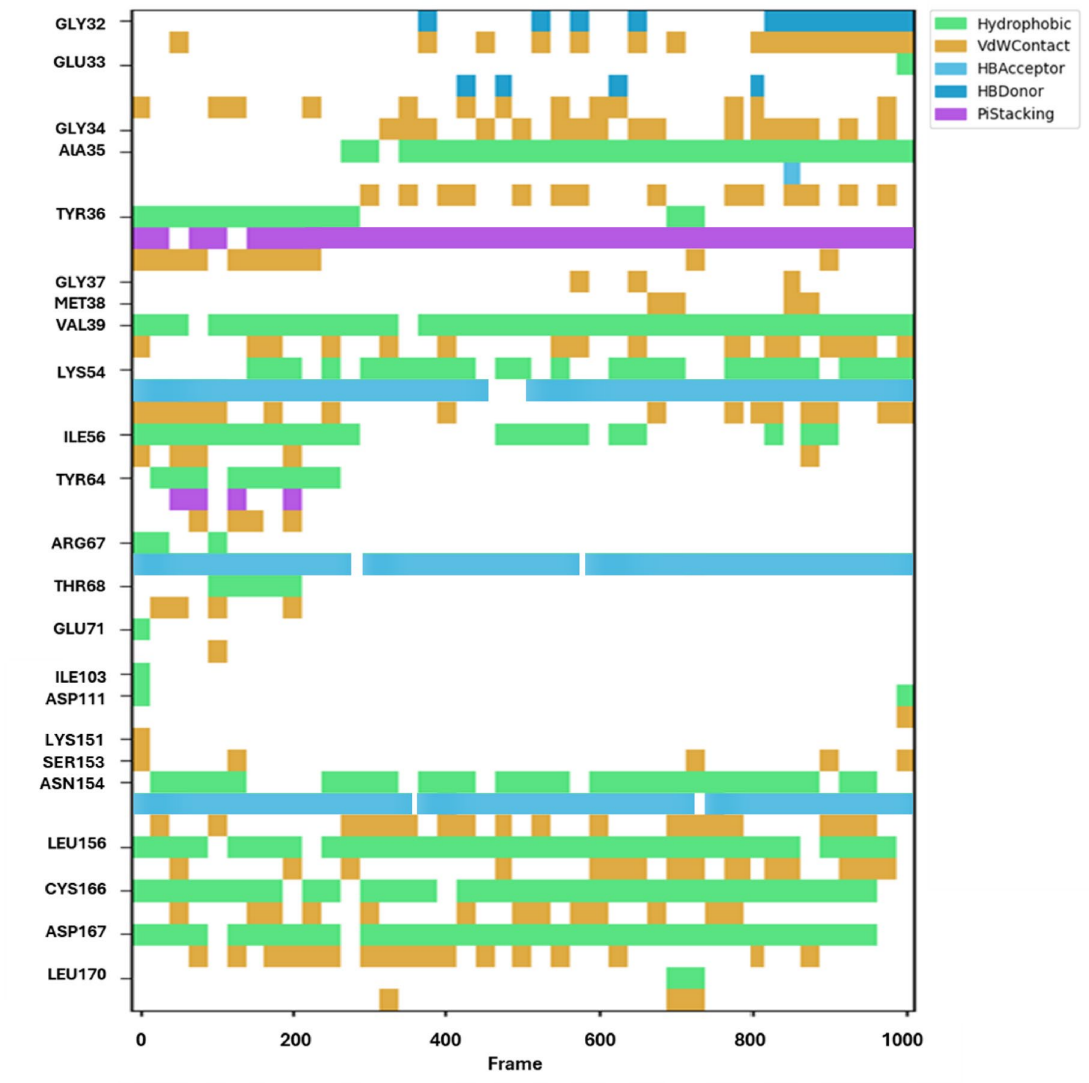
Various interacting amino acids with the sorts of interaction with **CPPI** into MAK1 protein active cavity.

It has been demonstrated that the binding contacts, including three stable H-bonding and several hydrophobic interactions, were steady during the MD trajectories of **CPPI** simulation with the MAPK1 receptor Fig. 9. As seen, regular π-stacking with Tyr36, three hydrogen bonds with Lys54, Arg67, and Asn154 were found. The findings of MMPBSA’s computation of the complex’s different binding energies are shown in Tables 4 and S1. It demonstrates how the stability of the complex was maintained by these interactions.

**Table 4:** Free binding energies of **CPPI** with MAK1 protein in kJ/mol.

Binding energies	kJ/mol
Electrostatic energy	− 26.338 ± 0.479
Polar solvation energy	126.784 ± 0.837
SASA energy	− 15.937 ± 0.059
van der Waal energy	− 139.571 ± 0.579
$\Delta G$	− 55.103 ± 0.578

## Conclusion

In this investigation, a new coumarin compound **(CPPI)** incorporated with pyridylpyrazolyl-indolin-2-one moiety was synthesized and evaluated for its antimicrobial and wound healing efficacy. **CPPI** showed considerable antibacterial activity, against the pathogenic *Staphylococcus aureus* MRSA, the resilient *Bacillus cereus*, and the formidable *Pseudomonas aeruginosa*. The target compound significantly promoted the migration of skin fibroblast cells and stimulated the wound healing process in both in vitro and in vivo experiments. Moreover, the treatment facilitated complete re-epithelialization of the wounds as well as the formation of well-structured granulation tissue and reduced signs of wound infection. Molecular docking studies of **CPPI** towards the key proteins involved in skin wound healing indicated higher binding affinity against COX-2 along with a stable complex during molecular dynamics simulations.

## Materials and methods

### Chemistry

Melting points were measured using an uncalibrated Electrothermal IA 9000 device. FTIR transmittance spectra were obtained using Vertex 80 FTIR spectrometer from Brucker (Germany), using KBr pellet method. A JEOL JMS-700 instrument (Tokyo, Japan) was employed to obtain high-resolution mass (HR-TOF-ESI-MS) spectral data for the target compound. NMR analysis, including ^1^H and ^13^C NMR spectra, was conducted on Bruker 500 NMR spectrometers at the Faculty of Pharmaceutical Science, Tokushima Bunri University, Japan. Chemical shifts were expressed in *δ* (*ppm*), with coupling constants reported in Hz. Reaction progress was monitored *via* TLC using silica gel aluminum sheets 60 F254 (Merck) with a chloroform/methanol (9.8:0.2 v/v) eluent and iodine-potassium spray. Compounds **2**, **4** and **5** were synthesized according to a previous method^45^. Characterization data of the newly synthesized compounds are provided in the supplementary materials.

#### Synthesis of 3-(3-(4-hydroxy-2-oxo-2 ***H*-chromen-3-yl)-5-(pyridin-3-yl)-1*H***-pyrazol-1-yl) indolin-2-one (CPPI)

Compound **5** (1 mmol) reacted with isatin (1 mmol) in 10 mL of absolute ethyl alcohol. The reaction mixture was subjected to reflux for a duration of 10 h. Following the cooling phase, the resultant product was isolated *via* filtration and subsequently washed with ethyl alcohol and air dried to obtain the target compound, designated as **CPPI**.

Buff powder, m.p. 291–292 °C, yield (85%). IR (KBr, cm^−1^): 3437 (OH), 3077 (NH), 1722, 1709 (C = O). ^1^H NMR (500 MHz, DMSO-d_6_): δ = 6.29 (s, 1H, CH-indole), 6.95 (d, 1H, *J* = 8.0 Hz, Arom-H), 7.01 (t, 1H, *J* = 7.5 Hz, Arom-H), 7.26 (d, 1H, *J* = 7.5 Hz, Arom-H), 7.32 (s, 1H, CH-pyrazole), 7.33 (t, 1H, *J* = 8.0 Hz, Arom-H), 7.38 (t, 1H, *J* = 7.0 Hz, Arom-H), 7.44 (d, 1H, *J* = 7.5 Hz, Arom-H), 7.58–7.61 (m, 1H, Arom-H), 7.67–7.71 (m, 1H, Arom-H), 7.88–7.91 (m, 1H, Arom-H), 8.13 (d, 1H, *J* = 6.5 Hz, Arom-H), 8.74 (d, 1H, *J* = 4.5 Hz, Arom-H), 8.89 (s, 1H, H2-pyridine), 10.88 (s, 1H, NH), 13.14 (s, 1H, OH). ^13^C NMR (126 MHz, DMSO) δ = 61.12, 95.38, 107.36, 110.81, 115.51, 116.84, 122.77, 124.12, 124.40, 124.96, 125.07, 125.37, 125.65, 130.56, 133.74, 137.36, 143.04, 143.51, 147.98, 149.83, 151.02, 152.56, 160.13, 163.61, 173.34.TOF-ESI-MS: [M + Na] ^+^: Calcd for C_25_H_16_N_4_NaO_4_ 459.1069; found 459.1069.

### Biological assays

#### Antimicrobial activity screening

The antimicrobial effectiveness of the newly synthesized compound was evaluated using the agar cup plate diffusion method. This involved testing **CPPI** compound against various pathogens, including Methicillin Resistant *Staphylococcus aureus* MRSA ATCC (Gram-positive bacteria), *Bacillus cereus* (Gram-positive bacteria) ATCC, and Carbapenem-resistant *Pseudomonas*,* aeruginosa* CRPA ATCC (Gram-negative bacteria) along with fungal pathogens such as *Candida albicans* ATCC and *Aspergillus niger* ATCC. Bacterial pathogens were pre-activated in nutrient broth medium (Condalab, Spain) at 37 °C for 24 h, while fungal pathogens were activated in Potato Dextrose Broth (Condalab, Spain) at 28 °C for 48 h under shaking conditions. The inoculum size for each pathogen was standardized using serial dilution and quantified by Colony Forming Unit (CFU) to maintain a consistent pathogen concentration throughout the tests. Screening of **CPPI** compound against bacterial and fungal pathogens was performed using a fixed concentration of 20 µg/mL of **CPPI** compound along with standard antibiotics^36^. After incubation, the antimicrobial results were assessed by measuring the inhibition zone diameter (mm). To determine the Minimum Inhibition Concentration (MIC) and Minimum Bactericidal/Fungicidal Concentration (MBC/MFC) of **CPPI**, a stock solution was prepared and diluted to concentrations ranging from 5 to 100 µg/mL, following the standard broth microdilution method^36^. The MIC for each compound was initially determined using the turbidometry method and then confirmed by the CFU method. The MIC and (MBC/MFC) value of **CPPI** were defined as the lowest concentration that resulted in the minimum number of CFU compared to the untreated samples^46^.

#### In vitro wound scratch assay

The scratch assay was employed to measure the migration rates of human fibroblast (BJ) cells in order to assess the potential of the newly synthesized compound to promote wound healing. Cells, at a density of 2 × 10^5^, were plated in each well of a 24-well plate and cultured in complete RPMI medium containing 10% fetal bovine serum (FBS), 2 mM L-glutamine, and 1% penicillin-streptomycin, incubated at 37 °C with 5% CO_2_. After 24 h, the cells were starved in medium with 0.5% FBS for another 24 h. Following starvation, a sterile P200 pipette tip was used to scrape the monolayer of confluent cells horizontally. The debris was washed away with PBS. The cells were then treated with 12.5 µg/mL of **CPPI** compound, while untreated cells served as a negative control. Phase contrast images of the scratch, representing the wound, were taken at 0 h before the treatment using 4x magnification. After 24 h of incubation with **CPPI**, a second set of images was captured. The migration rate was determined by analyzing the images with ImageJ software, measuring the percentage of wound closure, and comparing it with the 0-hour value. An increase in the closed area indicated cell migration and demonstrated the wound healing potential of **CPPI** compound. All experiments were performed in triplicates and the data were calculated according to the following equation:

$${\mathrm{Wound}}\,{\mathrm{closure}}\,\left( \% \right)\, = \,\left( {{\mathrm{Measurement}}\,{\mathrm{at}}\,0\,{\mathrm{h}} - {\mathrm{Measurement}}\,{\mathrm{at}}\,{\mathrm{24}}\,{\mathrm{h}}} \right){\mathrm{/Measurement}}\,{\mathrm{at}}\,0\,{\mathrm{h}}\, \times \,{\mathrm{1}}00$$

#### The generation of in vivo wound healing model 

Healthy male Wistar Albino rats (*n* = 10), weighing between 180 and 200 g, were randomly divided into two groups. The control group (*n* = 5) received no treatment, while the second group (*n* = 5) was treated with **CPPI** compound, which was suspended in sterile water at a concentration of 25 mg/mL. A 200 µL dose of the suspension was applied to the wounds on days 0, 3, 5, 7, 10, and 14 post-wound creations. The rats were obtained from the animal facility at the Faculty of Pharmacy, Egyptian Russian University, Cairo, Egypt. The in vivo experiments were conducted in accordance with the ethical approval number (ERUFP-PC-24-005) granted by the Egyptian Russian University Research Ethics Committee. The in vivo wound healing model was generated as described in Abo-Salm et al. where the experiment was conducted over a two-week period^37^. Prior to the experiment, the rats were acclimatized for 10 days and kept under controlled environmental conditions, including regulated humidity and temperature, and a 12-hour light/dark cycle. Before inducing the wounds, the rats were anesthetized with isoflurane, and their dorsal hair was shaved with an electric razor. The wound area was disinfected with 70% ethanol, and full-thickness skin wounds were created using a 10 mm biopsy punch. A digital camera was used to capture immediate images of the wounds. Additional images of the wound area were taken over a two-week period on days 0, 3, 5, 7, 10, and 14 to monitor the healing process. On day 14, the wounds were excised under anaesthesia for histopathological examination. The wound area was calculated using ImageJ software. The area was determined by measuring the relationship between the image pixels and the corresponding length in millimetres. The in vivo experiments were performed in accordance with ARRIVE guidelines with an ethical approval from the Research Ethics Committee, Faculty of Pharmacy, Egyptian Russian University, Egypt, No. ERUFP-PC-24,005.

### Histopathological experiment

By the end of the experiment, the dissected wound and the surrounding tissue were washed in normal saline then immersed in neutral buffered formalin 10%. The samples were fixed for 24 h then transformed for paraffin technique. Firstly, the obtained samples undergo dehydration with ethyl alcohol in ascending grades then cleared in xylene, impregnated in soft paraffin then embedded and blocked in hard paraffin. By using of rotatory microtome the paraffin blocks were cut at 5 μm thickness. The obtained sections were mounted on clean and dry glass slides then stained with Haematoxylin and Eosin (H &E) stain and vascular endothelial factor stain. Finally, these stained sections were examined using LEICA (DFC290 HD system digital camera, Heerbrugg, Switzerland) connected to the light microscope using 10×, 20× and 40× objective lenses (Bancroft and Gamble, 2008). The histopathological examination was done in histopathology laboratory, department of histology, faculty of Veterinary medicine, Beni-Suef University.

### Molecular docking and molecular dynamics

The proteins tumor necrosis factor (TNF-α, PDB code: 2AZ5^47)^, arachidonate 5-lipoxygenase (LOX, PDB code: 6N2W^48)^, cyclooxygenases (COX-1, PDB code:2OYE^49^; COX-2, PDB code: 5IKR^50)^, and mitogen-activated protein kinases (MAPK1, PDB code: 4QTA^50^; MAPK3, PDB code: 4QTB^51)^ were used for the 3D crystal structure of target proteins. Marvin sketch^52^ was used to generate CPPI’s chemical structure, and the lowest conformation was used in molecular docking studies. Hydrogen atoms were added and non-protein moieties like water were removed using AutoDockTools^53^. In the current docking investigation, AutoDock Vina^54^ was chosen to predict protein-ligand interactions and binding affinities. The coordinates of the co-crystallized ligands were used to produce the grid boxes (Table S1). Discovery Studio Visualizer^55^ was used to analyze and display the docking data. Molecular dynamics (MD) computations were performed on the optimal docking pose to gain more information about the interaction stability of compound **CPPI** within the MAPK1 binding site, or protein-ligand stability. CHARMM-GUI solution builder^56,57^ was used to create the input files for MD calculations using the CHARMM force field parameters for MAPK1. CHARMM General Forcefield was used to generate the topologies of the chosen ligands using the CgenFF server. The input files were created using the same parameters as in our earlier study^58^ using the CHARMM-GUI solution builder and GROMACS 2024 was used to perform 100ns MD simulation for the complex and the apoprotein^59^. The trajectories of the MD simulations were analyzed using GROMACS tools. The gmmpbsa^60^, a GROMACS tool for estimating binding affinity, was used to perform MM/PBSA (Molecular Mechanics/Poisson−Boltzmann Surface Area) computations.

## Supplementary Information

Below is the link to the electronic supplementary material.

Supplementary Material 1

## Data Availability

All data generated or analyzed during this study are included in this published article and its supplementary information files.
