# Supplementary material for: Exploring the impact of the innovative compound 3-(3-(4-hydroxy-2-oxo-2H-chromen-3-yl)-5-(pyridin-3-yl)-1H-pyrazol-1-yl) indolin-2-one on accelerating wound recovery
Source: Sci Rep. 2026 Feb 21;16:7489. doi: 10.1038/s41598-026-37714-5 (PMC12929582; doi:10.1038/s41598-026-37714-5)
Supplement: Supplementary file 1 — Supplementary Material 1 [file 41598_2026_37714_MOESM1_ESM.docx]

**Supplementary material**

**Exploring the Impact of the Innovative Compound 3-(3-(4-Hydroxy-2-Oxo-2*H*-Chromen-3-yl)-5-(Pyridin-3-yl)-1*H*-Pyrazol-1-yl)Indolin-2-One on Accelerating Wound Recovery**

- ^1^H-NMR of compound **CPPI**.
- ^13^C-NMR of compound **CPPI**.
- HR-TOF-ESI-MS of compound **CPPI**.
- **Figure S1.** Antibacterial activity of the targeted molecule using agar-well diffusion.
- **Figure S2.** Overlay of the co-crystal ligand and **CPPI**.
- **Figure S3**. Docking of **CPPI** inside the active sites of MAPK3, TNF-α and LOX.
- **Figure S4**. Docking of **CPPI** inside the active site COX-1 and COX-2.
- **Figure S5**. MD simulations of compound CPPI.
- **Table S1**. Grid parameter for Docking studies of compound **CPPI**


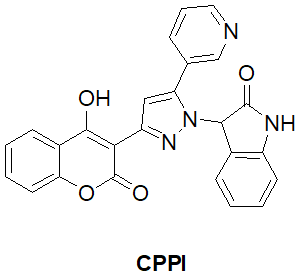


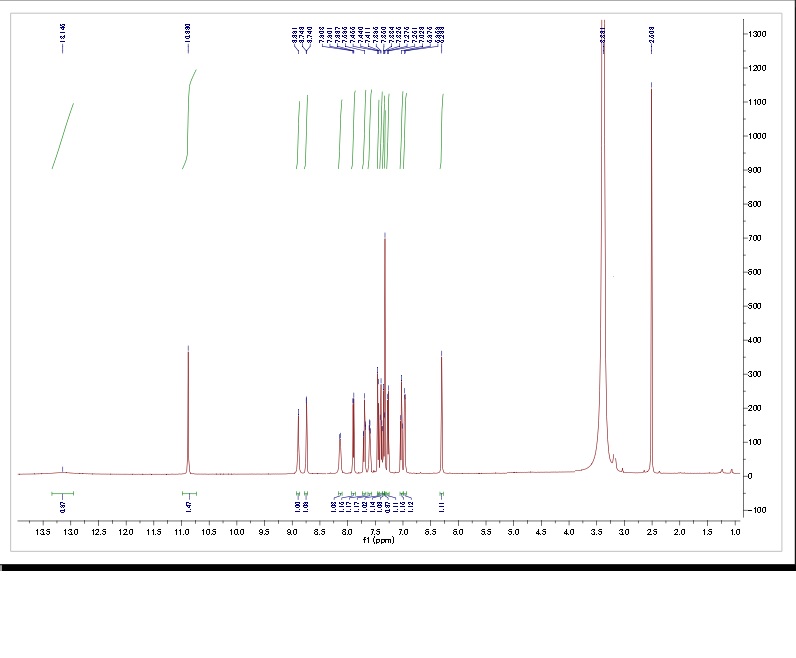


^1^H-NMR (500 Hz, DMSO) of compound CPPI.


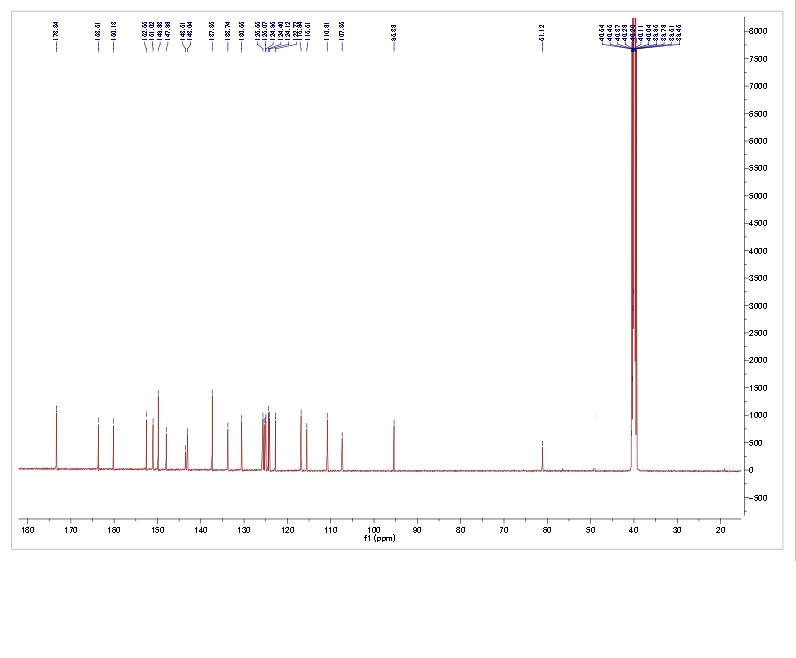


^13^C-NMR of compound CPPI.


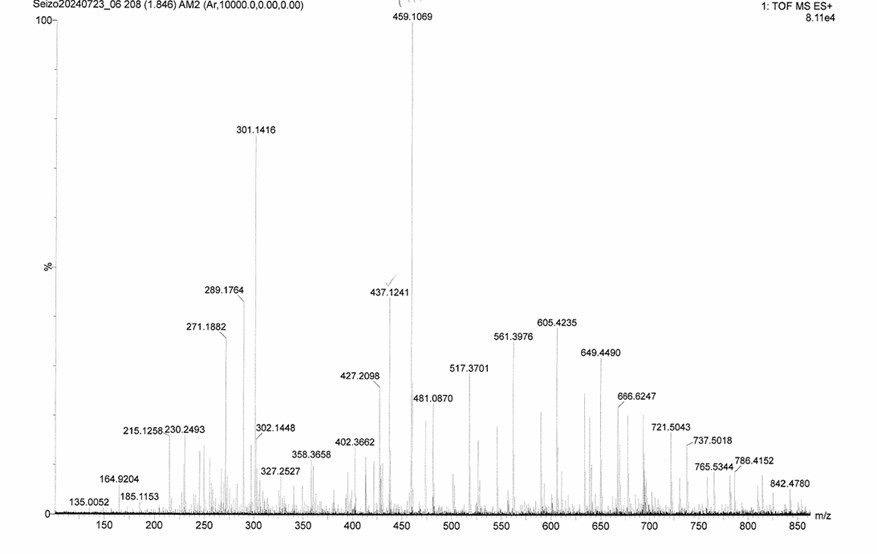


HR-TOF-ESI-MS of compound CPPI.

| ***Staphylococcus aureus* MRSA** | 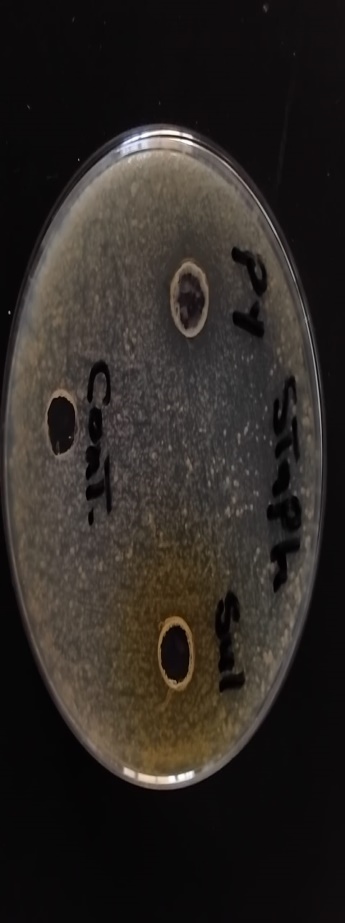 |
| --- | --- |
| ***Pseudomonas aeruginosa*** | 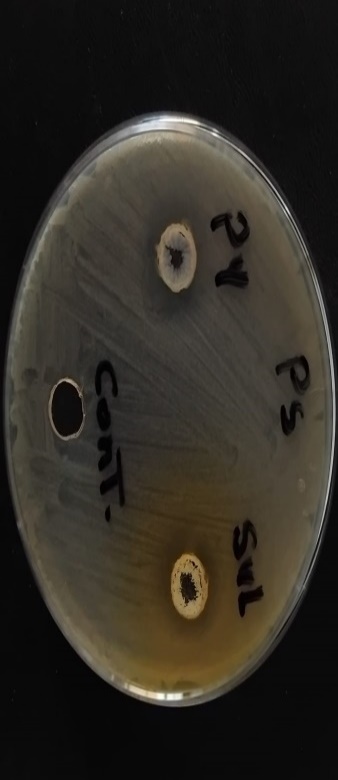 |
| ***Bacillus cereus*** | 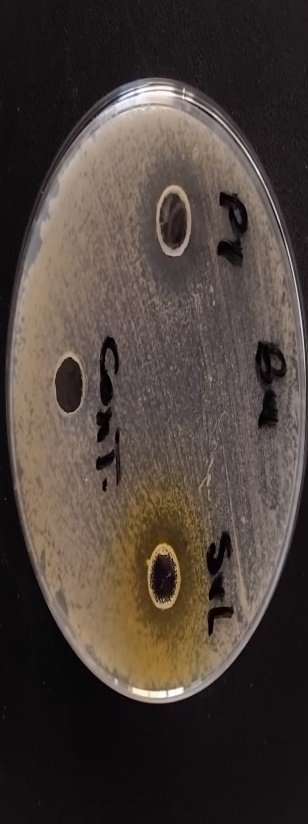 |
| ***Candida albicans*** | 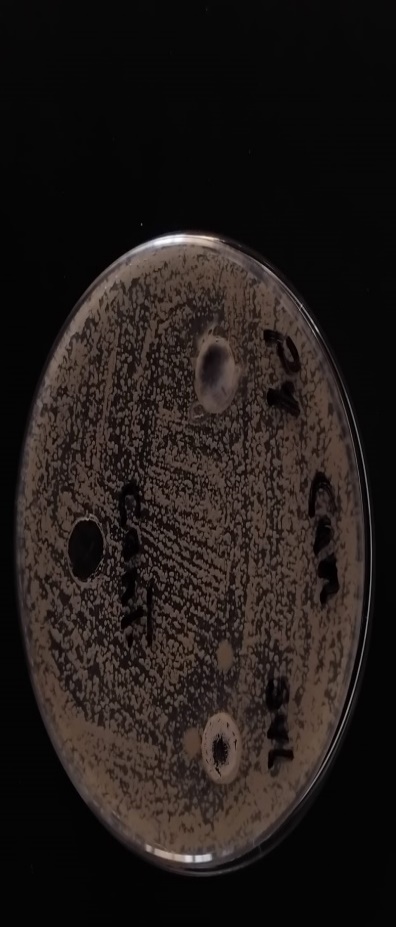 |
| ***Aspergillus niger*** | 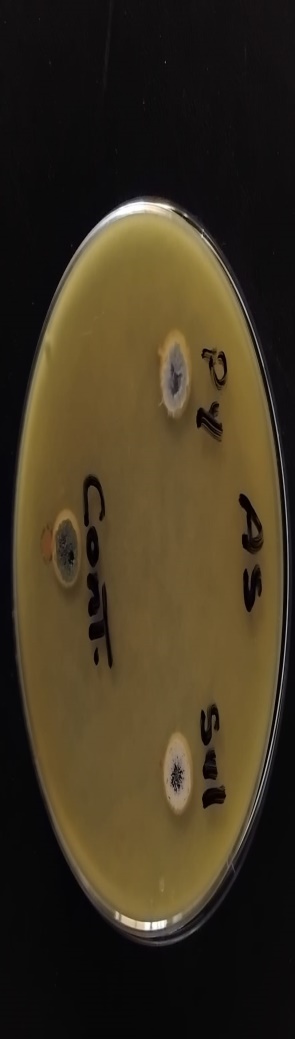 |

**Figure S1.** Antibacterial activity of the targeted molecule using agar-well diffusion.


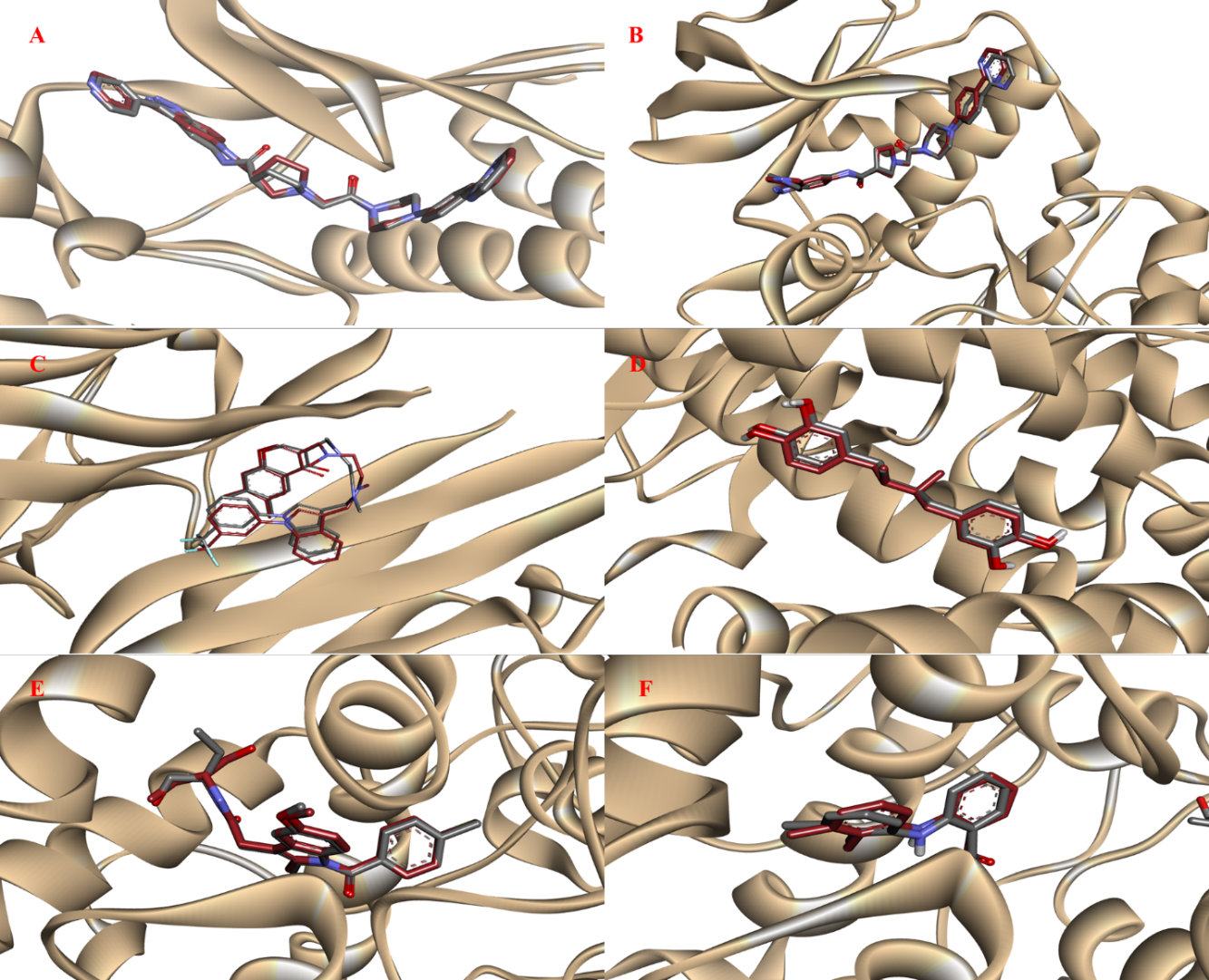


**Figure S2.** Overlay of the co-crystal ligand (red) and CPPI (A) MAPK1 “RMSD = 0.6199”, (B) MAPK3 “RMSD = 0.5230”, (C) TNF-α “RMSD = 0.9789”, (D) LOX “RMSD = 0.3476”, (E) COX-1 “RMSD = 1.3913” and (F) COX-2 “RMSD = 0.5341”.


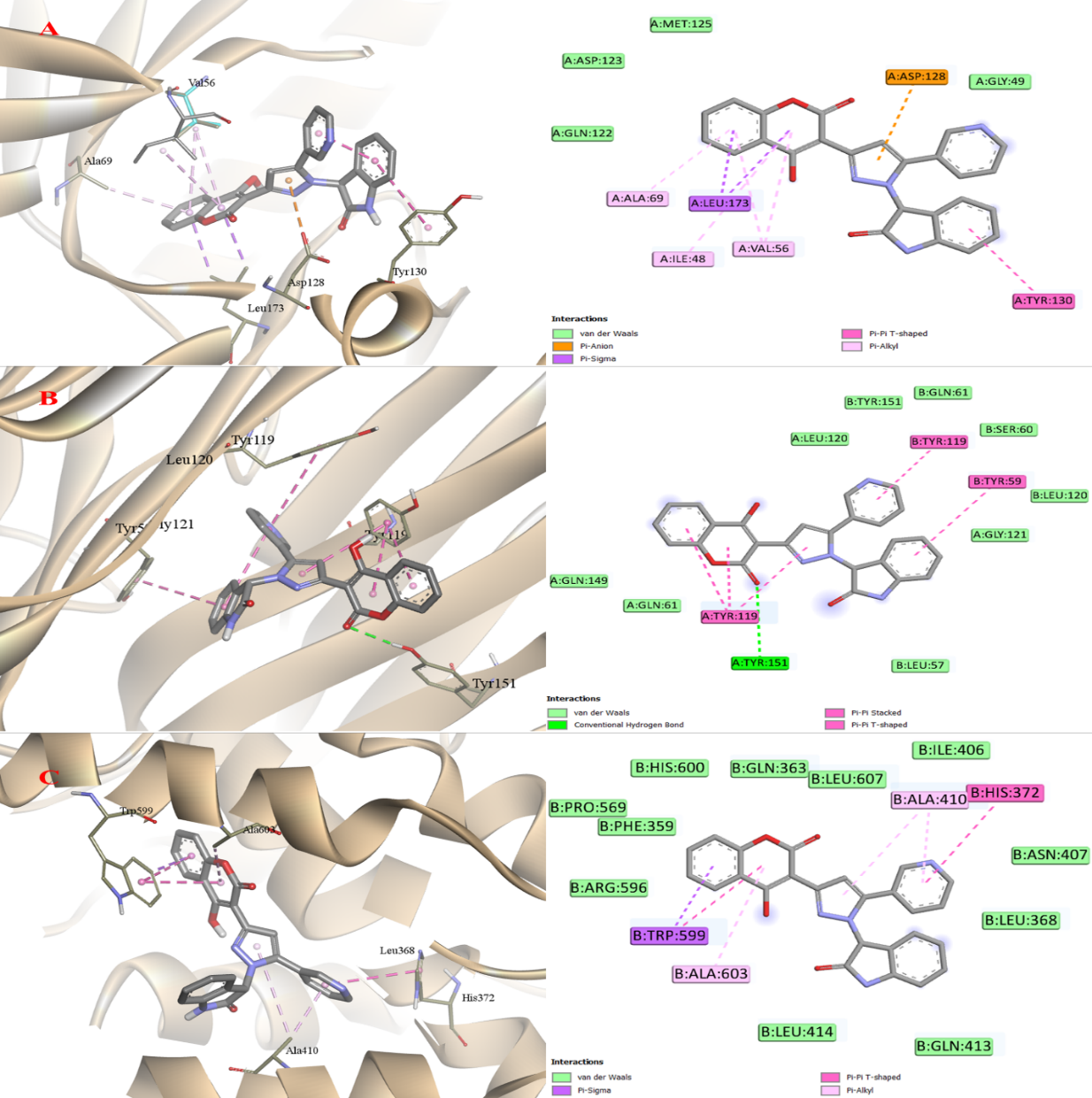


**Figure S3**. Docking of **CPPI** inside the active site of (A) MAPK3 (PDB = 4QTB), (B)TNF-α (PDB = 2AZ5), and (C) LOX (PDB = 6N2W).


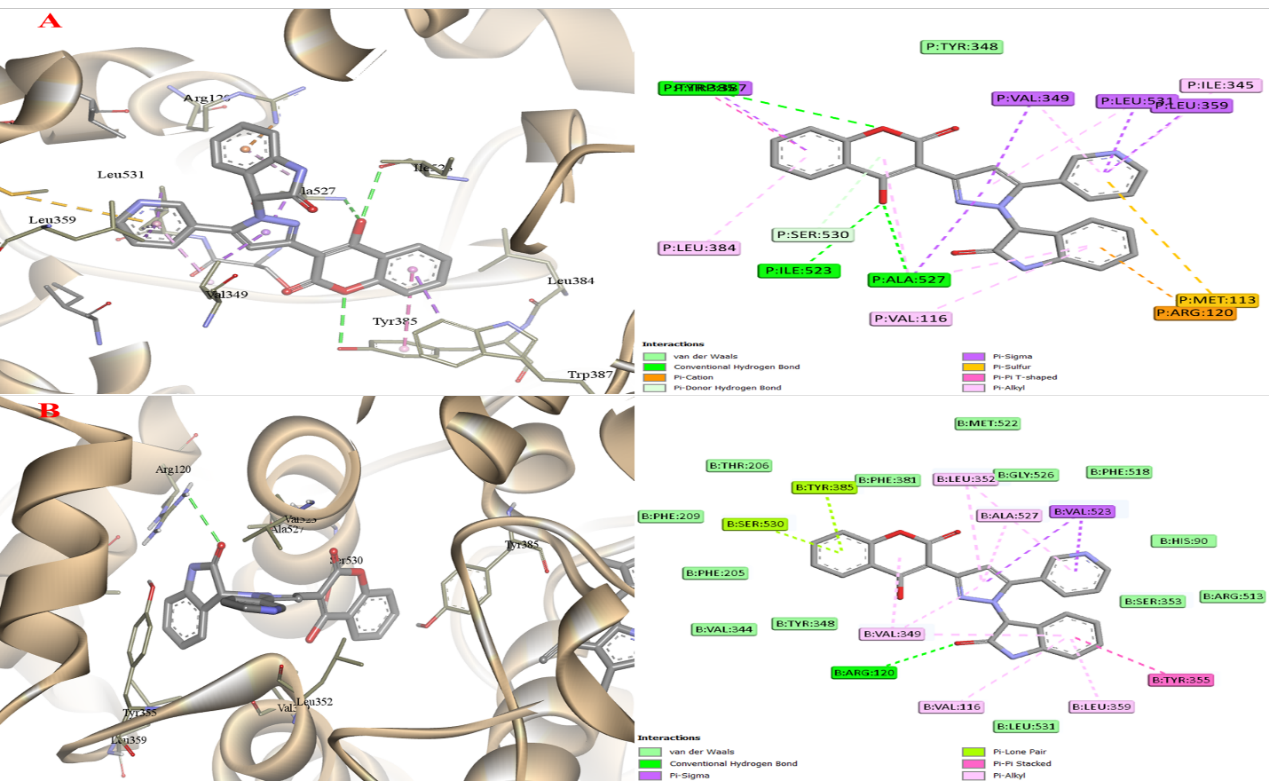


**Figure S4**. Docking of **CPPI** inside the active site of (A) COX-1 (PDB = 2OYE), and (B) COX-2 (PDB = 5IKR).


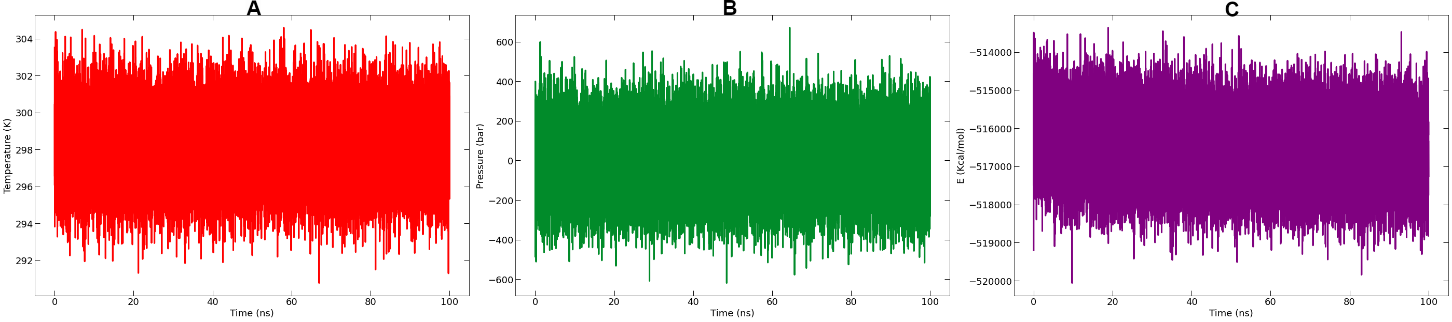


**Figure S5**. Plot of system parameters (A) Temperature, (B) pressure and (C) potential energy during the 100ns MD simulations

**Table S1**. Grid parameter for Docking studies

| Target protein | Grid parameter | |
| --- | --- | --- |
|  | Center (x, y, z) | Size (x, y, z) |
| MAPK1 | 27.9, 54.5, 35.2 | 21.6, 30.6, 16.5 |
| MAPK3 | 35.0, 56.7, 50.4 | 26.7, 20.4, 24.8 |
| TNFα | -19.2, 74.5, 33.8 | 19.1, 18.2, 18.5 |
| COX-1 | 250.2, 109.0, -40.8 | 19.2, 21.1, 18.4 |
| LOX | 35.9, 65.3, 38.3 | 14.5, 16.0, 24.0 |
| COX-2 | 40.7, 37.9, 86.4 | 20.0, 14.3, 16.9 |
